# Response to Chen et. al

**DOI:** 10.1186/s13690-022-00986-0

**Published:** 2022-11-16

**Authors:** Albert Stuart Reece, Gary Kenneth Hulse

**Affiliations:** 1 Division of Psychiatry, University of Western Australia, Crawley, WA 6009 Australia; 2 School of Medical and Health Sciences, Edith Cowan University, Joondalup, WA 6027 Australia

Whilst our paper was published in three parts to assist with readability it is essentially a single report. Our analysis moves logically from continuous and categorical bivariate studies to inverse probability weighted multivariate (IPWM) and then geospatiotemporal analysis and finally statistical analysis of geospatiotemporal models. Context is important. As stated by Chen and colleagues IPWM models are very powerful and present compelling conclusions and have the effect of transforming ecological into pseudo-randomized studies from which it is entirely proper to draw causal conclusions. That this report follows similar earlier IPWM and geotemporospatial reports on the commonest cancer in adults and women (breast cancer), the commonest cancer in children (acute lymphoid leukaemia) and total pediatric cancer and includes the commonest cancer in men (prostate cancer) forms a powerful backdrop for the present report (references in paper). We emphasized and now wish to re-emphasize that our analyses of prostate and ovarian cancer (both reproductive cancers) were exemplary and pathfinding since such detailed analyses have not previously been presented.

Importantly the results of the present study are closely concordant with analysis of similar European data [1].

Whilst Chen and co-workers erroneously invoke individual risk the focus of our paper is restricted to population-level risks. “Strength of association” – correlation—is the first of the Hill criteria of causation and is closely related to the “biological gradient”, the seventh criterion. Strength of association is quantified by E-values. The strong relationship between tobacco and lung cancer is a bivariate relationship with an E-value of nine. In analyzing any dataset it is obviously appropriate to look at the data themselves in this bivariate regard and this constitutes standard epidemiological practice. Our work neither validates nor confirms this approach but simply repeats it and openly presents the unprocessed data as is commonly undertaken for morbidity risk with tobacco, alcohol etc. It is unrealistic to expect all epidemiological series to present identical findings in relation to alcohol.

“Biological plausibility” the sixth Hill criterion is foundational and central to the causal argument. Whilst our paper presents many of these the stunning recent revelations of the longitudinal cannabis epigenomic study which included 810 hits across more than 20 different cancer types [2, 3] ushers in a whole new era in understanding the pleiotropic pathophysiology of diverse cannabinoids in cancer, aging and teratology.

Our list of covariates was the standard battery of substances, income and ethnicity used by many. Over-controlling for covariates is a major issue in this work as most covariates including income, education, ethnicity, other drug use and medication use are all themselves related to cannabis use and over-control can be expected to down-size the observed effect (as detailed in Judah Pearl’s “The Book of Why?” as referenced). We used IPW for measured covariates and effects of unmeasured covariates was quantified using E-values.

The rate of testicular cancer following cannabis was shown in meta-analysis at 2.59-fold [4]. If one assumes that the average age of significant exposure is about 20 years and the mean age of testicular cancer is 34 then this shortens the usual oncogenic incubation time from 34 to just 14 years or 2.4-fold. Multiplying this rate by the increased incidence shows a 6.3-fold increase in the oncogenic rate-incubation index. Similar observations apply to the cannabis-related pediatric cancers, acute myeloid and lymphoid leukaemias developing in the first few years of life and due to inherited genotoxicity. Evidently multiple decades are not necessarily required for cannabinoid carcinogenesis.

In particular the differentiation state of cells has recently been shown to be locked in place by a combined synchronous interlocking mechanism between the epigenome and the metabolome [5]. Cancer cells are uniformly de-differentiated and oncogenically transformed and commonly rely on glycolysis or glutaminolysis for their energy supply. This implies that the well known inhibition of mitochondrial metabolism by cannabinoids together with their widespread disruption of the epigenome and multiple oncogenic actions at once explains the links between cannabinoids and many different cancer types, their relatively rapid oncogenesis, and makes cannabinoid carcinogenesis an important experimental model for further research.

Cannabis is known to be toxic to actin and tubulin both directly proteomically and epigenomically and to disrupt the tubulin code and kinetochore function, properties which imply it disrupts and fractures the mitotic spindle and dislocates chromosomes thereby conferring indirect genotoxicity (clastogenicity) on cannabinoids as a class. For these reasons cannabinoids are implicated in chromothripsis (chromosomal shattering) which is not only a major engine of the genotoxicity in cancer, but is also very rapidly acting as the damage is so severe and extensive.

Our report mentions that the effects of cannabis to promote hepatocarcinogenesis occurs at concentrations 1,000 times lower than its anti-cancer effects. If it is true that cannabinoids have a bidirectional effect on carcinogenesis then epidemiology can indicate the net effect. Reports such as ours clearly indicate that the pro-cancer effects predominate and for all cannabinoids considered together have effects far exceeding that of tobacco and alcohol combined (Fig. 10 and Table 11 in Part 1).

In the same way that the present series of papers was a follow up and expansion of earlier reports so the present trilogy will in its turn be followed up and expanded upon in greater detail. We are delighted to report that the exhaustive and compendious IPWM, space–time, modelling and causal inferential covariate-specific encyclopaedic dissertation across many cancers implicitly suggested by Chen et.al. is progressing swiftly towards publication. Using methods now endorsed by Chen and co-workers the results are strongly confirmatory.

Finally, with divergent views held globally on the risks associated with cannabis use and many failing to identify conflict of interests, we must look clearly toward Science and academic rigour for answers.

## Data Availability

Not applicable.

## References

[1] Reece AS, Hulse GK, Preedy V, Patel V (2022). Cannabis Genotoxicity and Cancer Incidence: A Highly Concordant Synthesis of European and USA Datasets. Cannabis, Cannabinoids and Endocannabinoids.

[2] Schrott R, Murphy SK, Modliszewski JL, King DE, Hill B, Itchon-Ramos N, Raburn D, Price T, Levin ED, Vandrey R (2021). Refraining from use diminishes cannabis-associated epigenetic changes in human sperm. Environ Epigenet.

[3] Reece AS, Hulse GK (2022). Cannabis, Cannabidiol, Cannabinoids and Multigenerational Policy. Engineering.

[4] Gurney J, Shaw C, Stanley J, Signal V, Sarfati D (2015). Cannabis exposure and risk of testicular cancer: a systematic review and meta-analysis. BMC Cancer.

[5] Reece AS, Hulse GK (2022). Extending the "Paracentral Dogma" of biology with the metabolome: Implications for understanding genomic-glycomic-metabolic-epigenomic synchronization. Engineering.

